# Improved Discrimination of Influenza Forecast Accuracy Using Consecutive Predictions

**DOI:** 10.1371/currents.outbreaks.8a6a3df285af7ca973fab4b22e10911e

**Published:** 2015-10-05

**Authors:** Jeffrey Shaman, Sasikiran Kandula

**Affiliations:** Department of Environmental Health Sciences, Mailman School of Public Health, Columbia University, New York, New York, USA; Department of Environmental Health Sciences, Mailman School of Public Health, Columbia University, New York, New York, USA

## Abstract

Introduction: The ability to predict the growth and decline of infectious disease incidence has advanced considerably in recent years. In particular, accurate forecasts of influenza epidemiology have been developed using a number of approaches.

Methods: Within our own group we produce weekly operational real-time forecasts of influenza at the municipal and state level in the U.S. These forecasts are generated using ensemble simulations depicting local influenza transmission dynamics, which have been optimized prior to forecast with observations of influenza incidence and data assimilation methods. The expected accuracy of a given forecast can be inferred in real-time through quantification of the agreement (e.g. the variance) among the ensemble of simulations.

Results: Here we show that forecast expected accuracy can be further discriminated with the additional consideration of the streak or persistence of the forecast—the number of consecutive weeks the forecast has converged to the same outcome.

Discussion: The findings indicate that the use of both the streak and ensemble agreement provides a more detailed and informative assessment of forecast expected accuracy.

## Introduction

Recent applications of resampling and inference methodologies in the field of infectious disease modeling have led to the realization of real-time forecast by a number of research groups^1^^,^ 2 ^,^ 3 ^,^ 4 ^,^ 5 ^,^ 6 ^,^ 7 ^,^ 8 ^,^ 9. These approaches utilize, in varying combinations, statistical, dynamical or combined methods to predict future disease incidence.

Our group has been issuing real-time forecasts of influenza in the United States since the 2012-2013 season and posting these forecasts on a dedicated web portal^10^ since the 2013-2014 influenza season. Our approach to forecast presently centers on a combined dynamical model-inference system in which a compartmental model describing the propagation of influenza through a local population is optimized using data assimilation methods and real-time observations of influenza incidence. In practice, an ensemble of simulations is optimized from the past to the present, then integrated into the future to generate a real-time forecast. The optimization is performed using data assimilation, or Bayesian inference, methods, which constrain the model state variables and parameters to best represent the local outbreak as it has thus far manifested. The central idea is that if the ensemble of simulations can represent the outbreak as thus far observed, model-simulated forecasts will be more likely to represent future epidemic trajectories^3^^,^ 8 ^,^ 11 ^,^ 12. For influenza, these forecasts have been carried out at the municipal and state scale in the U.S.^10^.

Post-processing is an important means of organizing and presenting forecast information. As part of this post-processing, we use within ensemble variance, or other measures of ensemble spread, to provide a real-time estimate of the expected likelihood of a given forecast outcome^3^^,^ 11 ^,^ 12. For example, we can calculate the within ensemble variance of a particular peak timing forecast and then, by comparing that variance to those of past forecasts and their corresponding outcomes, quantify the likelihood that the real-time mean or mode prediction will be correct. In practice, we use past forecasts binned by lead time and variance to develop a look up table of expected accuracies. The upshot is a more discriminated prediction. For example, rather than simply predicting that influenza incidence will peak 4 weeks in the future with 2912 influenza cases per 100,000 patient visits, we can instead provide a prediction giving a 60% chance that an influenza outbreak will peak in 4 ±1 weeks and a 50% chance of peak incidence within ±25% of 2912 influenza cases per 100,000 patient visits.

These assigned probabilities, or expected accuracies, are analogous to numerical weather predictions in which, for example, there may be an 80% chance of precipitation tomorrow. A calibrated real-time forecast will, in the long run, be accurate in concordance with its expected accuracies. That is, over many decades, precipitation should occur on 80% of the days for which there had been a forecast of an 80% chance of precipitation tomorrow. Similarly, for influenza forecast, over many decades and locations, 60% of predictions issued with a 60% chance of influenza peaking in 4 ±1 weeks should peak in 4 ±1 weeks. Given the weekly frequency of forecasts, expected accuracy will need to be assessed over decades of prediction.

Real-time calibrated quantification of the expected accuracy of a forecast provides the end-user a more discriminated and actionable probabilistic prediction. For instance, a prediction of a 60% chance that influenza will peak in 4 ±1 weeks might elicit different response and mitigation procedures than a prediction of a 10% chance that influenza will peak in 4 ±1 weeks. To this end, it would be beneficial for the calibration of expected accuracy to be further discriminated and validated. Over the past two seasons only measures of ensemble variance and forecast lead time were used in our influenza forecast system^3^^,^ 11 ^,^ 12.

The epidemiological dynamics of influenza vary more slowly than weather, which varies considerably on daily to weekly time scales. There are typically only a handful of strains in circulation each season and only one or two isolated peaks of influenza incidence in a given locality. The time scale of variability for influenza is consequently much longer, which allows for longer accurate prediction lead times. It also means that successive weekly predictions of influenza at the same locality are not predictions of independent events.

In some instances, over successive weeks a municipal forecast may shift considerably due to changing observed dynamics and changing model constraint; however, in other instances, successive forecasts remain similar (Figure 1). This circumstance raises the following questions:

1) Does the week-to-week persistence of forecast outcomes provide further information on the expected accuracy of a prediction?

2) Does forecast accuracy increase as a function of this persistence?

Here, we explore these questions using 10 years of weekly seasonal influenza prediction for 95 cities and 50 states in the U.S. We find that inclusion of persistence in the assessment of forecast accuracy further discriminates probabilities for peak timing and peak intensity. For both outcomes accuracy increases with increasing persistence.

**Example real-time influenza forecasts generated during the 2014-2015 season using the SIRS-EAKF model-filter combination.**
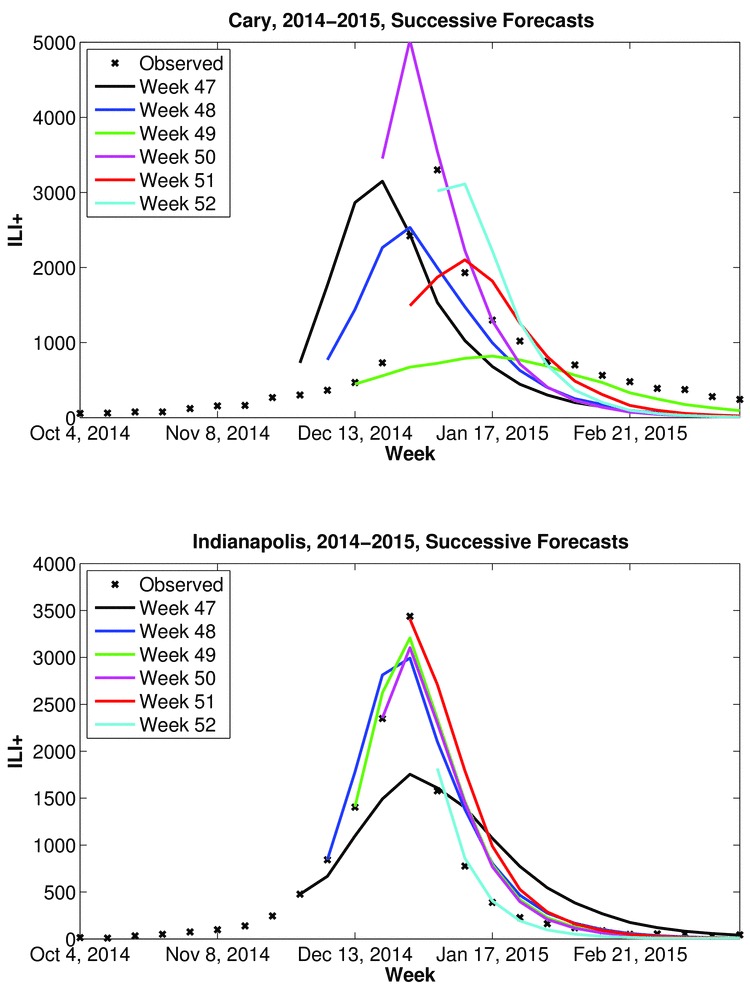
Successive weekly mean trajectory forecasts for November 30, 2014 (Week 47) through January 3, 2015 (Week 52) from 300-member simulations are shown for Cary, North Carolina (top) and Indianapolis, Indiana (bottom). Lines identify the ensemble mean forecast with colors indicating the week of forecast initiation. The ‘x’ mark weekly observations of ILI+. From week-to-week, the forecasts of peak timing and magnitude for Cary drift considerably, whereas for Indianapolis successive forecasts are more similar.

## Methods

We generated retrospective weekly municipal forecasts for 95 cities and 50 states in the U.S. during the 2003-2004 through 2014-2015 seasons. Due to the 2009 pandemic influenza outbreak, the 2008-2009 and 2009-2010 were excluded from this analysis. Forecast accuracy was then analyzed as a function of prediction lead time (i.e. how far in the future an event is predicted), ensemble variance (i.e. the within ensemble variance of the predicted metric), and prediction persistence (i.e. the number of successive weeks the same forecast outcome had been issued for a given location). Descriptions of the observations, models, data assimilation methods and analysis conducted are provided below.

**Influenza Incidence Data**

Weekly estimates of influenza incidence were generated by multiplying Google Flu Trend weekly estimates of municipal influenza-like illness (ILI)^13^ with census division regional weekly laboratory-confirmed influenza positive proportions as compiled by the U.S. Centers for Disease Control and Prevention (CDC) from National Respiratory and Enteric Virus Surveillance System (NREVSS) and U.S.-based World Health Organization (WHO) Collaborating Laboratories^14^. This combined metric, termed ILI+, provides a more specific measure of influenza incidence than ILI alone^3^^,^ 15.

**Epidemiological Models**

Four different models were used to generate forecasts. All four forms are perfectly-mixed, absolute humidity-driven compartmental constructs with the following designations: 1) susceptible-infectious-recovered (SIR); 2) susceptible-infectious-recovered-susceptible (SIRS); 3) susceptible-exposed-infectious-recovered (SEIR); and 4) susceptible-exposed-infectious-recovered-susceptible (SEIRS). The differences among the model forms align with whether waning immunity, which allows recovered individuals to return to the susceptible class, or an explicit period of latent infection (the exposed period) is represented.

As the SEIRS model is the most detailed, we present it here. All other forms are derived by reduction of these equations, which are as follows:

\begin{equation*}\small{\frac{dS}{dt}=\frac{N-S-E-I}{L}-\frac{\beta{(t)}IS}{N}-\alpha}\end{equation*} (1)

\begin{equation*}\small{\frac{dE}{dt}=\frac{\beta{(t)}IS}{N}-\frac{E}{Z}+\alpha}\end{equation*} (2)

\begin{equation*}\small{\frac{dI}{dt}=\frac{E}{Z}-\frac{I}{D}}\end{equation*} (3)

where *S* is the number of susceptible people in the population, *t* is time in years, *N* is the population size, *E* is the number of exposed people, *I* is the number of infectious people, *N-S-E-I* is the number of recovered individuals, \begin{equation*}\small{\beta{(t)}}\end{equation*} is the contact rate at time *t*, *L* is the average duration of immunity, *Z* is the mean latent period, *D* is the mean infectious period, and \begin{equation*}\small{\alpha}\end{equation*} is the rate of travel-related import of influenza virus into the model domain.

The contact rate, \begin{equation*}\small{\beta{(t)}}\end{equation*}, is given by \begin{equation*}\small{\beta{(t)}=R_0(t)/D}\end{equation*} , where \begin{equation*}\small{R_0(t)}\end{equation*}, the basic reproductive number, is the number of secondary infections the average infectious person would produce in a fully susceptible population at time *t*. Specific humidity, a measure of absolute humidity (AH), modulates transmission rates within this model by altering \begin{equation*}\small{R_0(t)}\end{equation*} through an exponential relationship similar to how AH has been shown to affect both influenza virus survival and transmission in laboratory experiments^16^:

\begin{equation*}\small{R_0(t)=R_{0min}+(R_{0max}-R_{0min})e^{-aq(t)}}\end{equation*} (4)

where \begin{equation*}\small{R_{0min}}\end{equation*} is the minimum daily basic reproductive number, \begin{equation*}\small{R_{0max}}\end{equation*} is the maximum daily basic reproductive number, \begin{equation*}\small{a=180}\end{equation*}, and \begin{equation*}\small{q(t)}\end{equation*} is the time-varying specific humidity. The value of a is estimated from the laboratory regression of influenza virus survival upon AH^17^. Simulations were performed with fixed travel-related seeding of 0.1 infections per day (1 infection every 10 days).

Results from all 4 models are presented, as these are the models we have been using operationally to generate real-time forecasts 10. We use the four model forms, as no single model has consistently outperformed the others.

**Specific Humidity Data**

Specific humidity data were compiled from the National Land Data Assimilation System (NLDAS) project-2 dataset. These gridded data are available in hourly time steps on a 0.125° regular grid from 1979 through the present^18^. Local specific humidity data for each of the 95 cities and 50 states included in these forecasts were assembled for 1979-2002 and averaged from hourly to daily resolution. As daily AH is itself highly variable from year-to-year, we then constructed a 1979-2002 (24 year) daily climatology for each city and state. These smoother time series, which represent average AH conditions for each day of the year for a specific locality, were used as the daily location-specific AH forcing during both model optimization and forecast.

We restricted the AH data to 1979-2002, as this time span precedes the period being retrospectively forecast, i.e. we do not employ data that would not have been available for real-time, operational forecast. Both the AH and ILI+ raw data are posted for download at cpid.iri.columbia.edu .

**Data Assimilation**

Three ensemble filter methods—the ensemble Kalman filter^19^, the ensemble adjustment Kalman filter^20^ and the rank histogram filter^21^—and a particle filter (PF) with resampling and regularization^22^ were used in conjunction with ILI+ to optimize the compartmental models prior to forecast. These four filters are currently used in our operational real-time influenza forecasts^10^. Ensemble filter simulations were run with 300 ensemble members while the PF simulations, which require more particles to span state variable and parameter space effectively^12^, were run with 10,000 particles.

The three ensemble filter algorithms are used iteratively to update ensemble model simulations of observed state variables (i.e. influenza incidence) to better align with observations (i.e. ILI+). These updates are determined by halting the ensemble integration at each new observation, computing the Kalman gain using that new observation and the distribution of current model states (the prior), and then using the Kalman gain to calculate a posterior for the observed state variables. Cross ensemble co-variability is used to adjust both the unobserved state variables and model parameters. The posterior is then integrated to the next observation and the process is repeated. Through this iterative updating process the ensemble of simulations provides an estimate of the observed state variable (i.e. influenza incidence), as well as the unobserved variables and parameters (e.g. susceptibility and mean infectious period).

Generally, Kalman filters assume normality of both the likelihood and prior distributions during an update. Differences among the ensemble filter algorithms manifest in the means by which the update is specified. The ensemble Kalman filter (EnKF) is a stochastic, perturbed observation form of the Kalman filter in which the update of each ensemble member is computed using the current observation plus Gaussian random noise^23^. That is, the posterior for each ensemble member is simply the weighted sum of the prior for that ensemble member and the observation plus random noise with variance equal to the observational error variance (OEV). The weights themselves are calculated as ratios of the ensemble prior variance and the OEV. The OEV itself is the estimated error associated with a given ILI+ observation and is defined as in previous works^3^^,^ 11.

The ensemble adjustment Kalman filter (EAKF) employs a deterministic algorithm to compute the ensemble posterior mean and variance^20^. At each update, the EAKF algorithm aligns the first two ensemble posterior moments with those predicted by Bayes theorem.

Unlike the EnKF and EAKF, the rank histogram filter (RHF) does not impose a Gaussian structure on the prior, observations and posterior^21^; rather, this filter employs an algorithm that creates an approximate probability distribution by ordering (i.e. ranking) the ensemble prior. In this fashion, the RHF admits non-Gaussian distributions, thus relaxing the normality assumption inherent to most Kalman filters.

For all three ensemble filters, multiplicative inflation^11^^,^ 20 was applied following the assimilation of each weekly observation of ILI+. The inflation was used to counter the ensemble filter tendency toward ‘filter divergence', which occurs when the prior ensemble spread becomes spuriously small. In the absence of inflation, the system may give too little weight to the observations and thus diverge from the true trajectory.

Unlike the above ensemble filters, PFs are an alternate class of assimilation method that do not require assumptions about linearity or normality. The PF approach used here adopts sequential importance sampling with resampling and regularization^22^^,^ 24. Resampling generates a new suite of particles with equal weight during the model integration whenever the effective sample size is low. Regularization jiggles the state and parameter values of each resampled particle to eliminate particle redundancies and further sample parameter space around each previously highly weighted particle. As a consequence of resampling and regularization, a much richer range of parameter and state space is spanned than with a basic PF- which relies only on the initial parameter choices.

For all 16 model-filter combinations a scaling factor was employed to convert ILI+ from number of influenza cases per 100,000 patient visits, to influenza incidence, the quantity represented in the compartmental models, per Shaman et al.^3^. Additional details on the application of the ensemble filters and PF to infectious disease models are provided in Shaman and Karspeck^11^ and Yang et al.^12^.

**Forecasts**

The recursive updating of model state space variables and parameters, carried out by the filtering process, is meant to align these characteristics to better match the observed epidemic trajectory of influenza incidence. The premise is that if the model can be optimized to represent observations as they have thus far manifested, it stands a better chance of generating a forecast consistent with future conditions.

In practice for each city and season, we initiated our model simulations and data assimilation on Week 40 of the calendar year. Each week, the ensemble of simulations (300 for the ensemble filters, 10,000 for the PF) was updated using a given filter. All 16 combinations of the 4 models (SIR, SIRS, SEIR, SEIRS) and 4 filters (EnKF, EAKF, RHF and PF) were run, and the accuracy of each individual ensemble forecast was assessed separately. For weeks 45-64 of the flu season, following assimilation of the latest ILI+ observation, the posterior ensemble was integrated into the future to generate a forecast for the remainder of the season.

Initial parameter values for all runs were chosen randomly from the following uniform ranges: \begin{equation*}\small{R_{0max}}\end{equation*} ~ U[1.3, 4]; \begin{equation*}\small{R_{0min}}\end{equation*} ~ U[0.8, 1.2]; *Z* ~ U[1, 5 days]; *D* ~ U[1.5, 7 days]; *L* ~ U[1, 10 years]. Initial state variable values *I*(0) and *S*(0) were chosen randomly from a distribution of October 1 state values generated through 100,000 31-year free simulations of the SIRS compartmental model forced with 1972-2002 daily AH for New York State (see Shaman et al.^16^ and Shaman and Karspeck^11^, for details). *E*(0) initial values were also randomly chosen from the *I*(0) distribution. For all runs the population size, N, was 100,000. As ILI+ observations are estimates of the number of patients with influenza per 100,000 patient visits and are multiplied by a scaling factor^3^ for assimilation as per capita influenza incidence in the models, the population size is arbitrary and allows for use of a common scaling across all sites. To account for the stochastic effects of the randomly-chosen initial conditions, each model-filter combination was run 5 separate times for each location, thus generating 5 separate forecasts for each week and location.

**Analysis of Expected Accuracy**

We assessed the effect of forecast persistence on expected accuracy for outbreak peak timing and magnitude. We quantified persistence or ‘streak’ as the number of consecutive weeks for which the forecast during a season in a given locality had not changed (within ±1 week for peak-week timing, ±25% for peak intensity). We then assessed peak timing and magnitude forecast accuracy as a function of streak, within ensemble variance and forecast lead (i.e. how far in the future the event is predicted). Note that forecast lead is not measured relative to the observed peak week, as in real time this observation is unknown; rather, it indicates when, relative to the week of forecast initiation, the model mean trajectory predicts the peak week will occur.

Retrospective out-of-sample expected accuracy predictions were made using 10-fold cross validation. In each of the 10 iterations (one per fold), 90% of the 16 model-filter combination forecasts were used to quantify the percentage of predictions correct (within ±1 week for peak-week timing, ±25% for peak intensity) and a look-up table of these percentages as a function of forecast lead, streak and/or ensemble variance (binned in 0.5 increments) was generated. The expected accuracy of the remaining 10% of the forecasts were then assigned, based on this look-up table. The process was repeated without replacement so that out-of-sample expected accuracy predictions were generated for all forecasts.

To test whether the use of persistence adds value in assigning expected accuracy:

a) We calculated the Spearman rank correlation between the observed errors and accuracy estimates calculated using ensemble variance alone and using a combination of ensemble variance and persistence. We expect to see a stronger correlation with the latter if persistence is beneficial.

b) We performed a Student’s T-test of expected accuracies comparing correct and incorrect forecasts. With a valid assignment of expected accuracies, the mean expected accuracy of correct forecasts should be greater than the mean expected accuracy of incorrect forecasts. Moreover, if persistence is beneficial, the difference in means should be greater when a combination of both ensemble variance and persistence is used.

The analysis of peak timing and intensity accuracy includes weekly forecasts initiated after the observed peak of the season has passed. Such continued assessment of the forecasts is critical, as, when run in real time, identification of the observed peak is not straightforward because: 1) a second, larger observed peak of incidence may subsequently develop; and 2) the model may predict spurious future peaks. Consequently, it is necessary to continue generating and evaluating predictions after the peak appears to have occurred.

## Results

Correlations of forecast absolute error (e.g. a prediction of peak timing is off 3 weeks) versus expected accuracy are presented in Table 1. That is, the table provides a comparison of the magnitude of the forecast error versus the forecast expected accuracy; a larger negative correlation indicates that forecast error decreases with increasing expected likelihood. For predictions of both peak timing and intensity there is a stronger negative correlation when both variance and streak are used to discriminate expected accuracy than when either variance or streak are used alone.

**Table 1. Spearman rank correlation of forecast error versus forecast expected accuracy.** Larger negative correlations indicate that forecast error decreases as forecast expected accuracy increases. All correlations are significant at p < 1e-6.

	Variance	Streak	Variance & Streak
Peak Timing	-0.639	-0.599	-0.661
Peak Intensity	-0.629	-0.588	-0.643

Student’s T-tests show that there are statistically significant (p < 1e-6) differences between the mean expected accuracies assigned to correct and incorrect forecasts (Table 2). For peak timing, the difference is highest, 32.64 percentage points, when both ensemble variance and streak are used as discriminators. Similarly, for peak intensity the difference is greatest when both variance and streak are used to discriminate accuracy: 30.06 percentage points. When only predictions made prior to the peak are considered the difference in means decreases to 9.64 and 3.19 percentage points for peak timing and peak intensity, respectively, but remains statistically significant (p < 1e-6). These findings indicate that higher expected accuracy is assigned on average to forecasts that indeed prove to be correct and the inclusion of persistence is beneficial.

**Table 2. Percentage point differences in the mean expected accuracy of forecasts that proved accurate versus those that did not.** The values in brackets show the mean expected accuracy of those correct and incorrect forecasts. All differences positive indicating higher mean expected accuracy for the correct forecasts and are statistically significant by Student’s t-test at P < 1e-6.

	Variance	Streak	Variance & Streak
Peak Timing, All Forecasts	30.17 [63.33, 33.16]	29.03 [62.73, 33.70]	32.64 [64.61, 31.97]
Peak Timing, Forecasts Pre-Peak	8.62 [33.54, 24.92]	4.48 [30.52, 26.05]	9.64 [34.28, 24.63]
Peak Intensity, All Forecasts	32.55 [67.54, 34.99]	32.07 [67.31, 35.24]	33.97 [68.21, 34.24]
Peak Intensity, Forecasts Pre-Peak	3.39 [31.43, 28.04]	0.77 [29.57, 28.80]	3.90 [31.78, 27.88]

Figures 2 and 3 present heat maps of expected accuracy as a function of forecast lead, streak and ensemble variance for peak timing and peak intensity, respectively. Expected accuracy rises as the observed peak moves from the future to the past (from negative forecast leads to positive forecast leads). In addition, expected accuracy rises as streak increases and ensemble variance decreases. The effect of streak is clearer for peak timing (Figure 2).

**Heat map showing the expected accuracy of peak timing forecasts as a function of forecast week, streak and within ensemble log variance:**
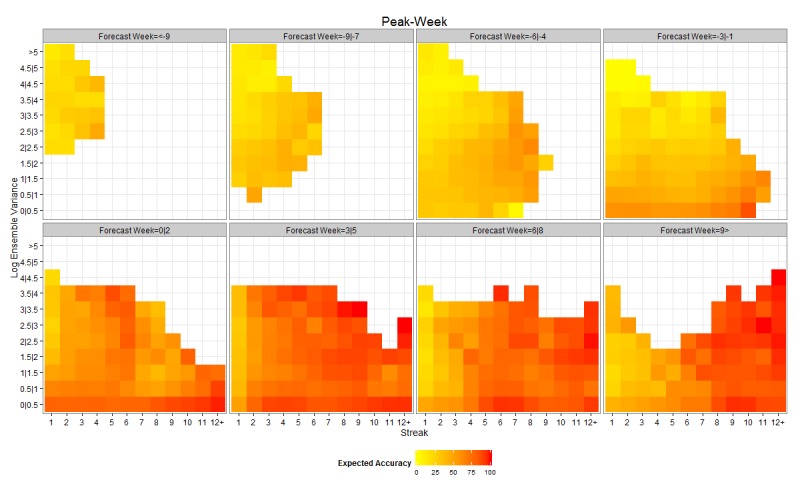
The 8 subplots present groupings for forecast week (i.e. when the peak is predicted relative to the current week) with negative numbers indicating the future and positive numbers the past.

**As for Figure 2, but for peak intensity.**
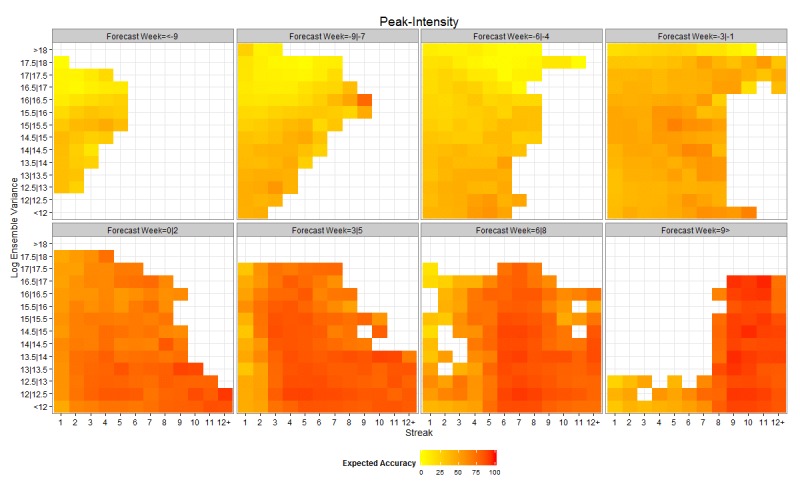

By distinguishing the forecasts by lead, streak and ensemble variance, expected accuracy is further discriminated. Peak timing expected accuracy rises to as high as 85% for 1-3 week forecast leads and 62% for 4-6 week forecast leads (Figure 2). Similarly, peak intensity expected accuracy is as high as 66% for 1-3 week forecast leads and 57% for 4-6 week forecast leads (Figure 3). It is worth noting that positive lead forecasts, those predicting the peak has already occurred, while higher than negative lead forecasts, do not reach 100% accuracy. This finding is not surprising as the models may forecast the peak has passed and fail to anticipate a second, larger surge of influenza incidence.

Figures 4 and 5 present plots of expected accuracy versus observed frequency segregated by forecast lead, streak and ensemble variance for peak timing and peak intensity, respectively. Both figures demonstrate that these out-of-sample predictions are well calibrated, i.e. the observed frequency matches expected accuracy. For example, forecasts of peak timing made 1-3 weeks prior to the actual peak with ensemble log variance between 0.5 and 1 and a streak of 6 weeks have an expected accuracy of ~60% and were observed to be correct ~60% of the time. This calibration indicates that the expected accuracies are a reliable measure of forecast certainty. Furthermore, for peak timing there is a clear increase of expected accuracy as streak increases and variance decreases.

**Calibration plots of observed frequency as a function expected accuracy for forecast peak timing.**
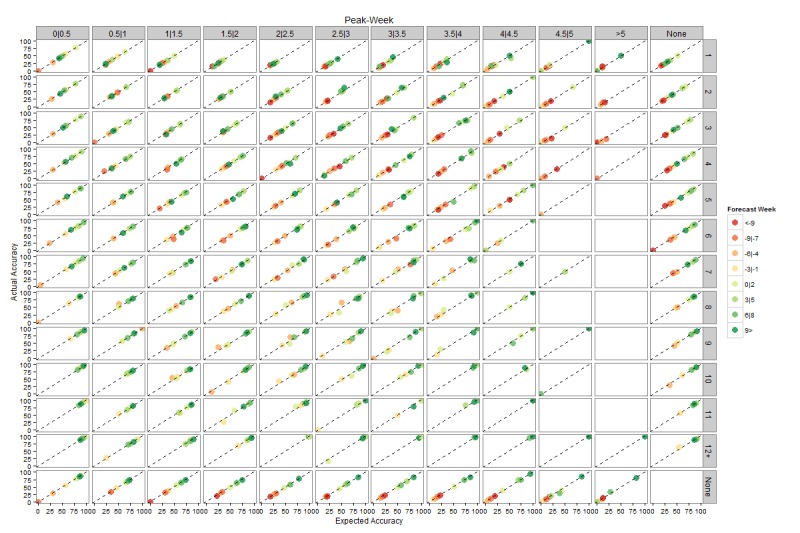
These plots are segregated by forecast week, streak and within ensemble log variance. A calibrated forecast will fall on the diagonal line (e.g. forecast outcomes with a 70% expected accuracy have an observed frequency—are observed accurate—70% of the time). The ‘None’ column presents forecasts only discriminated by streak; the ‘None’ row presents forecasts only discriminated by ensemble variance.

**As for Figure 4, but for peak intensity.**
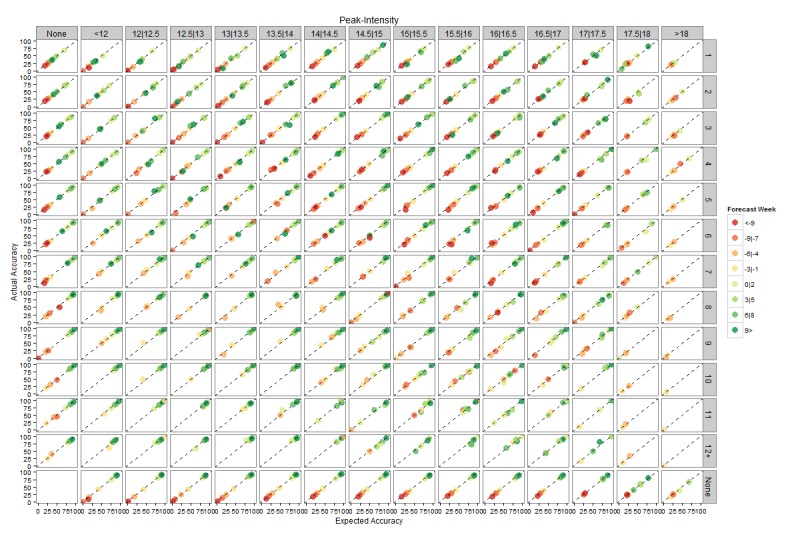

## Discussion

Our findings indicate that use of forecast streak, in addition to forecast lead and ensemble variance, further discriminates the accuracy of our influenza predictions. This discrimination can be applied in real-time so that operational forecasts of influenza incidence are more precisely segregated. By better distinguishing good and bad forecasts, our predictions are not only well calibrated (i.e. reliable), but they are also ‘sharp’--that is, outcomes are associated with more precise certainties.

This idea of sharpness is important. It is the ability to distinguish high and low probability events. As a counter example, one can predict a 50% chance that a coin flip will yield heads. Over many predictions, those forecasts will prove reliable but not sharp (nor particularly informative). For influenza prediction we want to avoid this regression to the mean. That is, rather than assign average probabilities over all predictions, our post-processing aims to distinguish accurate and inaccurate forecasts. While we still want overall forecast accuracy to improve, we also want to be able to reliably discriminate high certainty predictions (e.g. 70%, 90%) from lower certainty events (e.g. 10%, 30%).

Such discrimination allows individuals and public health officials to act on forecast information with greater confidence. For example, a forecast of, e.g., a 10% chance that influenza will peak in 5 ±1 weeks indicates that the best model estimate is a peak in 5 ±1 weeks but also signals that there is considerable disagreement among those ensemble model predictions and/or lower persistence across weeks; consequently, there is a low probability that this outcome will be observed. In contrast, a forecast of an 80% chance that influenza will peak in 5 ±1 weeks indicates that there is considerable agreement among the predictions and/or a long streak, and consequently a high probability that the outcome will be observed.

Moving forward, we will use forecast streak to help discriminate the expected accuracy of our real-time operational influenza forecasts^10^. This calibration approach might also be applied to other recurrent infectious diseases with lengthy observational records.

## Competing Interests

JS discloses consulting for JWT and Axon Advisors and partial ownership of SK Analytics. SK declares no competing interests.
